# Severe Diabetic Ketoacidosis as the Initial Presentation of m.3243A>G‐Related Mitochondrial Diabetes in a 19‐Year‐Old Woman

**DOI:** 10.1111/1753-0407.70268

**Published:** 2026-08-30

**Authors:** Rathana Ly, Toshihiro Kobayashi, Kensaku Fukunaga, Takanobu Saheki, Takafumi Yoshimura, Eri Harada, Keisuke Takahashi, Seisuke Sato, Wenyi Jiang, Haotian Zhang, Hitomi Imachi, Koji Murao

**Affiliations:** ^1^ Department of Endocrinology and Metabolism, Faculty of Medicine Kagawa University Miki‐cho Kagawa Japan

## Abstract

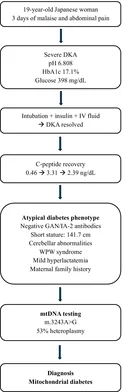

Dear Editor‐in‐Chief,

1

Thank you very much for your kind suggestion to resubmit the manuscript as a Letter to the Editor. We have revised and condensed the manuscript in accordance with the Editor's suggestion, including citation of the specified article.

Diabetic ketoacidosis (DKA) is generally considered uncommon in mitochondrial diabetes because endogenous insulin is often partly preserved [1, 2, 3]. DKA has been reported, although it appears to be an uncommon presentation of mitochondrial diabetes. Keidai et al. reported DKA in a patient with previously diagnosed mitochondrial diabetes during infectious enteritis, while Ohwada et al. indicated DKA following glucocorticoid exposure, accompanied by transient lactate elevation during intravenous insulin therapy [4, 5]. More recently, Zhou et al. reported a 36‐year‐old man, previously diagnosed with type 2 diabetes, who subsequently developed recurrent DKA and seizures, and was later diagnosed with MELAS and mitochondrial diabetes due to the m.3243A>G variant [6].

We encountered a 19‐year‐old Japanese woman with no previous diagnosis of diabetes who presented with severe DKA. Her plasma glucose was 398 mg/dL, HbA1c: 17.1%, pH: 6.808, bicarbonate: 1.0 mEq/L, PCO2: 6.7 mmHg, base excess: −38.8 mEq/L, and lactate: 21 mg/dL, which required intensive care and mechanical ventilation. Islet‐related autoantibodies were negative. Although serum C‐peptide was low during the acute phase (0.46 ng/mL), it subsequently recovered to 3.31 and 2.39 ng/mL after metabolic stabilization, indicating substantial residual endogenous insulin secretion. These findings were atypical for classic autoimmune type 1 diabetes. The total ketone body concentration was 8964 μmol/L and rapidly improved to 64 μmol/L after fluid resuscitation and insulin therapy. She was subsequently transferred from the intensive care unit and discharged on Day 14.

Several extra‐pancreatic findings prompted reconsideration of the diagnosis. She was markedly short (141.7 cm), had childhood Wolff‐Parkinson‐White syndrome, cerebellar atrophy, and an old infarct‐like cerebral lesion on magnetic resonance imaging, persistent mild hyperlactatemia, and a maternal family history suggestive of mitochondrial disease. Genetic testing identified the mtDNA m.3243A>G variant with 53% heteroplasmy. Thus, severe DKA was the initial clinical presentation that led to recognition of m.3243A>G‐related mitochondrial diabetes. Figure 1 summarizes the clinical course and diagnostic evaluation leading to the diagnosis of mitochondrial diabetes.

**FIGURE 1:**
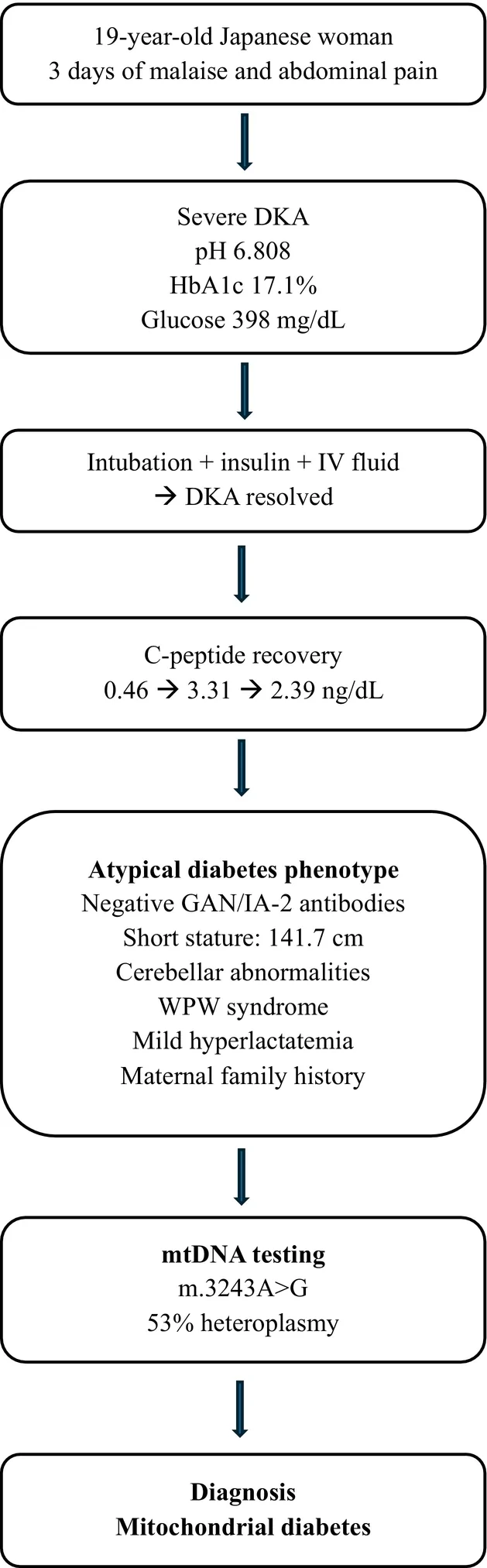
Clinical course and diagnostic evaluation leading to the diagnosis of m.3243A>G‐related mitochondrial diabetes.

The report by Zhou et al. recently described a 36‐year‐old man with recurrent DKA, seizures, and MELAS features and the m.3243A>G variant [6], but our case adds a distinct clinical scenario with two clinically important respects: the patient was only 19 years old, and DKA was the initial manifestation of previously unrecognized diabetes rather than a complication arising after diabetes had already been diagnosed.

This case highlights that in young patients presenting with DKA, negative islet autoantibodies, recovery of C‐peptide secretion, short stature, neurological or cardiac abnormalities, mild lactate elevation, or a maternal inheritance pattern should prompt consideration of mitochondrial diabetes. Early recognition of these features may facilitate genetic diagnosis and family screening, anticipatory surveillance for multisystem complications, and long‐term management.

## Author Contributions

R.L. conceived the report, analyzed the clinical data, performed the literature review, and drafted the manuscript. T.K. contributed to the study conception, clinical interpretation, and revision. K.M. supervised the study, interpreted the clinical findings, critically revised the manuscript for important intellectual content, and approved the final version. K.F., T.S., T.Y., E.H., K.T., S.S., W.J., H.Z., and H.I. participated in the patient's clinical management, contributed to data interpretation, and critically reviewed the manuscript. All authors meet the International Committee of Medical Journal Editors (ICMJE) authorship criteria and approved the final manuscript.

## Funding

The authors have nothing to report.

## Ethics Statement

The study was approved by the Ethics Committee of the Faculty of Medicine, Kagawa University (ethical approval number: 2026‐111).

## Consent

Written informed consent for publication was obtained from the patient.

## Conflicts of Interest

The authors declare no conflicts of interest.

## Data Availability

The data that support the findings of this study are available on request from the corresponding author. The data are not publicly available due to privacy or ethical restrictions.
